# Is There Association Between Stress and Bruxism? A Systematic Review and Meta-Analysis

**DOI:** 10.3389/fneur.2020.590779

**Published:** 2020-12-07

**Authors:** Victória dos Santos Chemelo, Yago Gecy de Sousa Né, Deborah Ribeiro Frazão, Renata Duarte de Souza-Rodrigues, Nathalia Carolina Fernandes Fagundes, Marcela Baraúna Magno, Cláudia Maria Tavares da Silva, Lucianne Cople Maia, Rafael Rodrigues Lima

**Affiliations:** ^1^Laboratory of Functional and Structural Biology, Institute of Biological Sciences, Federal University of Pará, Belém-Pará, Brazil; ^2^Faculty of Medicine and Dentistry, University of Alberta, Edmonton, AB, Canada; ^3^Department of Pediatric Dentistry and Orthodontics, School of Dentistry, Federal University of Rio de Janeiro, Rio de Janeiro, Brazil

**Keywords:** stress, bruxism, systematic review, meta-analysis, association

## Abstract

This systematic review and meta-analysis aimed to investigate a possible association between stress and bruxism in humans. This study was conducted according to Preferred Reporting Items for Systematic Reviews and Meta-Analyses (PRISMA) guidelines under the code CRD42020188862, and the searches were performed on the following databases: PubMed, Scopus, Web of Science, Cochrane, LILACS, OpenGrey, and Google Scholar. This systematic review evaluated observational studies in adult humans with and without stress to verify the association between bruxism and the presence of stress. The risk of bias was evaluated through the Joanna Briggs Institute Critical Appraisal Tools for Analytical Cross-Sectional Studies. In quantitative analysis, the Odds Ratio (OR) and their 95% confidence interval (CI) were calculated through a fixed-effect model. Furthermore, a summary of the overall strength of evidence was presented using the Grading of Recommendations, Assessment, Development, and Evaluation (GRADE). A total of 1,458 studies were identified, and six were included in this systematic review. Two studies included were classified with a low risk of bias, and the others were classified with a moderate risk of bias. In three articles, a meta-analysis was performed and showed an association between these two factors (OR 2.07 [1.51, 2.83], *p* < 0.00001, *I*^2^ = 45%). Besides that, a low certainty of the evidence was detected among this association. Stressed individuals show a higher chance of presenting bruxism when compared to healthy individuals. Despite the low heterogeneity found in the quantitative analysis among the articles reporting an association between stress and bruxism, further studies with similar methods are necessary to understand this relationship better.

## Introduction

Stress can be defined as “a condition or feeling experienced when a person perceives that the demands placed on them exceed the resources the individual has available” (1). Bad, excessive, or prolonged stress reactions may exceed the organism's natural adaptive capacity and permanently affect stress responses (2). The impact of stress on physiological and psychological processes is determined by characteristics of the stress stimulus (3), being able to trigger changes in several functions in the organism, including repercussions on the stomatognathic apparatus (4).

Bruxism is a repetitive muscular activity of the jaw characterized by grinding or clenching the teeth and bracing or thrusting of the mandible, is mainly regulated centrally, and may involve more than dental contact (5). Currently, bruxism has a distinction between sleep bruxism and awake bruxism. Sleep bruxism is a sleep-related movement disorder characterized as rhythmic or non-rhythmic of masticatory muscle activity (5, 6). In contrast, awake bruxism is a non-functional behavior during wakefulness characterized by repetitive or sustained tooth contact and/or by bracing or thrusting of the mandible (5, 6). However, both forms are associated with different personal behaviors as potential clinical consequences (5).

The factors associated with the development of bruxism are bad habits, such as smoking, high alcohol, and coffee consumption (7); sleep apnea syndrome, anxiety disorder, depression, respiratory diseases (8–10). Recent studies show that emotional changes may be associated with bruxism. However, few studies investigate these isolated manifestations (11).

This systematic review study was developed to gather evidence in the literature to answer the question: “Is there evidence in the literature that points to a possible association between stress and bruxism in humans?”

## Materials and Methods

### Protocol and Registration

This systematic review was registered at PROSPERO under the registration number CRD42020188862 and performed according to Preferred Reporting Items for Systematic Review and Meta-Analysis (PRISMA) checklist (12) (Supplementary Table 1).

### Eligibility Criteria and Search Strategy

The PECO strategy was followed in this systematic review. Observational studies in humans (P), with stress (E), and without stress (C) that verified the association between bruxism (O), were included. Also, opinion articles, case reports, descriptive studies, review articles, technical articles, guides, animal studies, and *in vitro* studies were excluded. No restriction about the diagnostic tool to assess the stress and age of the participants was applied. The null hypothesis of this review was: “There is no association between stress and bruxism.”

Searches were conducted in the following electronic databases, without language or year restriction until June 2020: PubMed, Scopus, Web of Science, The Cochrane Library, LILACS. The gray literature was searched through OpenGrey and Google Scholar. The search strategy was prepared to be held in PubMed and contained a combination of controlled pre-defined MeSh and free terms related to stress and bruxism. This strategy was adapted according to the syntax rules of each database. Boolean operators (OR, AND) were used to combine searches (Supplementary Table 2).

After searches, all relevant citations were saved in a bibliographic reference manager (EndNote, x9 version, Thomson Reuters). Duplicated results were considered only once. The titles and abstracts that did not adhere to the established eligibility criteria were excluded. The resulting articles were evaluated and judged by their full text.

Additional citations were sought from the analysis of the reference list of all articles previously selected. The selection process was conducted by two examiners (VC and YN) and checked by a third examiner (RL), in cases of disagreements.

### Data Extraction and Studies Selection

Data extraction was carried by two examiners (VC and YN), independently. A third reviewer was consulted in case of disagreement.

It was took into consideration information related to the author, local and year of publication, study design, sample source and sample sizes, age of participants, stress and bruxism evaluation method, statistical analysis, and main results (Table 1).

**Table 1:** Summary of characteristics and results of the included studies.

**Study design/ Country (Reference)**	**Participants (Source of sample/sample size)**	**Age**	**Stress evaluation**	**Bruxism evaluation**	**Statistical analysis**	**Results**
Cross-sectional/Iran (13)	Isfahan Shahid Vatan Pour Airforce Base/(*N* = 172) 86: Presence of stress 86: Absence of stress	40 years	12-item General Health Questionnaire (GHQ-12)	12-item General Health Questionnaire (GHQ-12) and clinical examination	Chi-square test; Independent *t*-test and Pearson	Indicated a significant positive association between stress level and bruxism (*p* < 0.05).
Cross-sectional/Turkey (14)	Protective-care facilities in Kocaeli, Turkey and primary school, one elementary school and one high school in Kocaeli, Turkey/(*N* = 385) 184: Presence of stress 201: Absence of stress	8–18 years	Questionnaire about stressful life events	Questionnaire about bruxism (diurnal tooth grinding/clenching) and clinical examination	Chi-square, Spearman's correlation and Mann–Whitney *U*-tests	The mean number of oral parafuction (bruxism) was higher in children who reported emotional problems than in children who did not report any problems (*P* < 0.05)
Cross-sectional/Lithuania (15)	Department of Preventive and Pediatric Dentistry, Medical Academy, Kaunas, Lithuania/(*N* = 200) 171: Presence of stress 29: Absence of stress	15–19 years	Purpose-designed self-reported questionnaire; regarding stress experience; describe stress intensity and allocate into 10 stress intensity levels.	Questionnaire about systemic conditions (stress, GERD, bruxism)	ANOVA and chi-square test	Stress have an association with oral health status. The prevalence of bruxism increases with higher stress levels. The percentage of stress distribution among respondents with bruxism was detected statistically (*X*^2^ = 12.157; *p* = 0.002)
Cross-sectional/Brazil (16)	Brazilian Navy/(*N* = 486) 103: Presence of stress 383: Absence of stress	19–48 years	Stress symptoms inventory (SSI)	Extra and intraoral physical examination: Presence of wear facets using the Ordinal Wear Severity Scale Occlusal. Self-report of grinding bruxism sounds; painful sensitivity upon touching the masticatory muscles.	Chi-square test	The statistical analysis of the correlation between stress and bruxism was significant (*P* > 0.05).
Cross-sectional/Brazil (17)	Brazilian Police Officers/(*N* = 394) 180: Presence of stress 214: Absence of stress	Mean age: 35.5	Stress symptoms inventory (SSI)	Presence of wear facets on the anterior and/or posterior teeth. Score in accordance with the ordinal scale of wear severity. Grinding bruxism sounds; painful sensitivity upon touching the masticatory muscles.	Chi-square test	Emotional stress was associated with bruxism, independently of the type of work done by the police officer. The prevalence association between emotional stress in police officers and bruxism was statistically significant (*P* = 0.0004).
Cross-sectional/Israel (18)	Israel Air Force/(*N* = 57) 35: Presence of stress 22: Absence of stress	25.8 years ± 4.3 SD	Psychological questionnaires to assess two factors: Magnitude of workplace stress and Coping style.	Dental examination: tooth wear visually	Chi-square t- test, ANOVA following by *post hoc* comparisons.	The results of the questionnaires among pilots revealed a stress level of 3.84 (SD 0.54), whereas among non-pilots the stress level was 3.59 (SD 0.48). Association between work environment stressful demand and bruxism was clearly more noticeable among pilots (69%) than among non-pilots (27%).

In case of the absence of relevant information for data extraction or risk of bias evaluation, we attempted to contact the authors by email. A weekly email was sent to the authors for up to five consecutive weeks.

### Quality Assessment Analysis and Risk of Bias

The studies' quality and risk of bias were assessed through the Joanna Briggs Institute Critical Appraisal Tools for Analytical Cross-Sectional Studies (19). The tool consists of eight questions whose answers could be “yes,” “no,” “unclear,” and “not applicated.” The risk of bias was scored as Low when the study reached over 70% of the “yes” score, Moderate when the study reached from 50 to 69% of the “yes” score and High when the study reached up to 49% of “yes” score. Studies characterized as a “high risk of bias” were excluded (20, 21). The guidelines of evaluation criteria are described in Supplementary Table 3.

### Quantitative Synthesis (Meta-Analysis)

The quantitative synthesis to evaluate the relationship between stress and bruxism was assessed using Review Manager software v. 5.3. Number of Bruxism (events) and the total number of individuals in case (stressed) and control (none stressed) groups were included to calculate the Odds Ratio (OR) with a 95% confidence interval (CI). A fixed-effect model was used (22), and heterogeneity significance was evaluated using the I^2^ index. Thresholds for the interpretation of the I2 statistic were considered as suggested by Cochrane handbook (www.training.cochrane.org/handbook): 0–40%: might not be important, 30–60%: may represent moderate heterogeneity, 50–90%: may represent substantial heterogeneity, 75– 100%: considerable heterogeneity. A sensitivity analysis was performed to evaluate the influence of risk of bias in effect significance. During this phase, studies with some type of risk of bias were excluded from meta-analysis and changes in the overall significance were evaluated.

### Assessment of the Certainty of the Evidence

The certainty of the evidence (certainty in the estimates of effect) was determined for the outcome using the Grading of Recommendations Assessment, Development and Evaluation (GRADE) approach (23). Whereby observational studies start as low certainty in the body of evidence and could decreases to very low quality, if serious or very serious issues, related to the risk of bias, inconsistency, indirectness, imprecision, and publication bias, are present. Publication bias was evaluated through visual analysis of the funnel plot. Besides, the quality of the evidence can be upgraded if the magnitude of the effect is large or very large or if the effect of all plausible confounding factors would reduce the effect or suggest a spurious effect. The dose-response was not applied in the type of studies included in the present systematic review and as judged in a way to not uninfluenced in the final certainty of evidence. In this way, the quality of the evidence can vary from very low to high.

## Results

### Study Selection and Characteristics

1,458 articles were identified from all databases, and 548 duplicate citations were excluded. Titles and abstracts of 910 articles were verified following the entry criteria. 15 articles were selected for full-text appraisal, resulting in the exclusion of 895 articles. Six were included in this systematic review, and 3 of them were included in quantitative synthesis (meta-analysis). The articles excluded after reading in full and reasons for exclusions are shown in Supplementary Table 4.

### Results of Individual Studies

Among the six articles included, all of them were cross-sectional studies. An association between the stress and bruxism was reported in all six articles (13–18). In the case of the absence of relevant information for data extraction and risk of bias, the authors were contacted by email, were made to provide the lack of information (13). These studies were performed in Iran (13), Turkey (14), Lithuania (15), Brazil (16, 17), and Israel (18).

According to the study, the stress evaluation was performed through validated and not validated questionnaires, assessing life events and stress scale. The parameters used to evaluate bruxism by the selected studies were clinical features and/or questionnaires. The data extraction about the articles is in Table 1.

Three studies performed a clinical evaluation regarding bruxism evaluation, considering tooth wear, teeth clenching, and grinding as diagnostic parameters. A 6-point scale was considered for final diagnosis (13, 16–18). Four studies applied validated questionnaires to evaluate bruxism (13–15).

**Figure 1:**
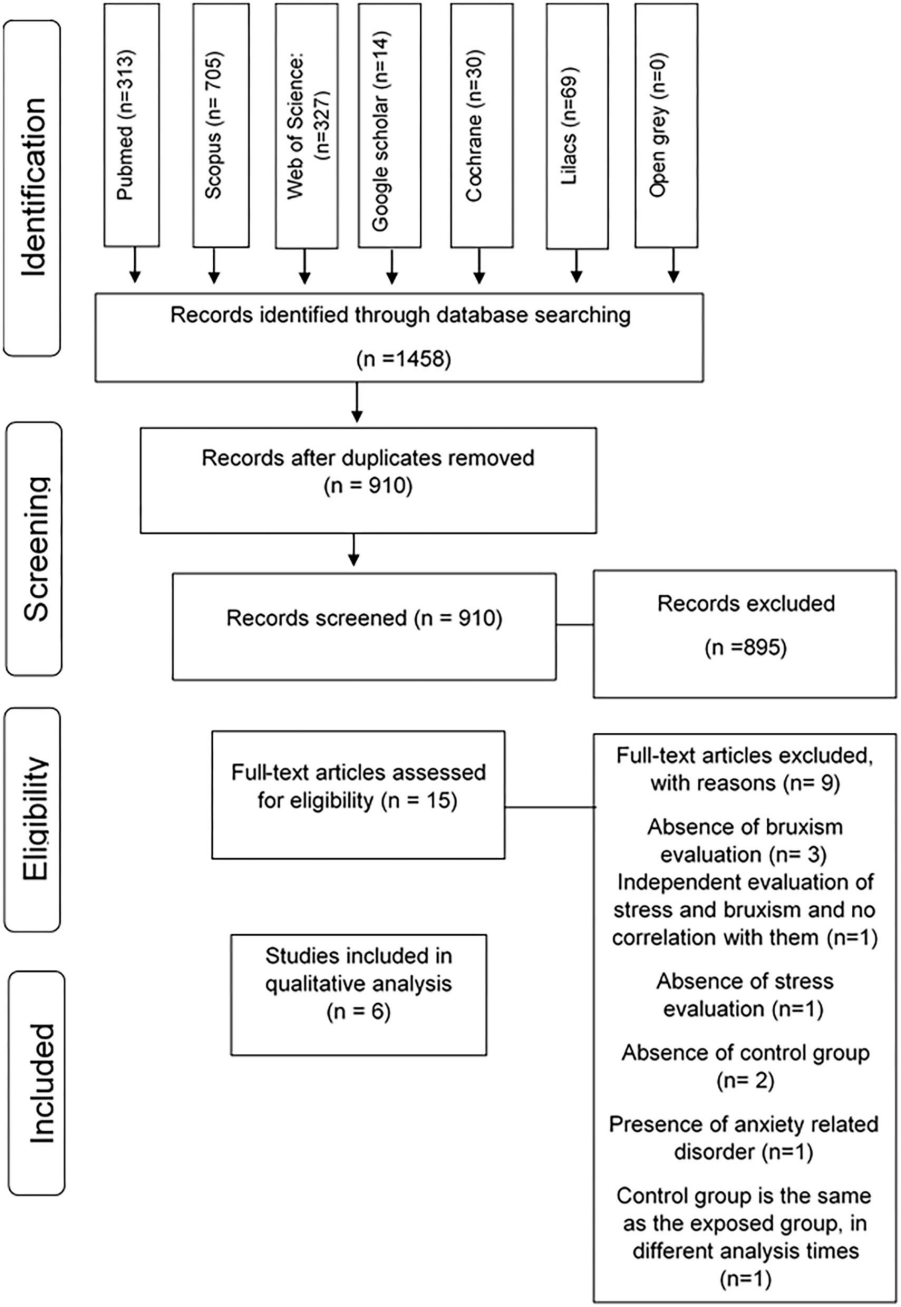
Flow diagram of databases searched according to PRISMA guidelines (Preferred Reporting Items for Systematic Review and Meta-Analysis).

Six articles were considered for quality assessment, and their summary selection is shown in Figure 1.

### Qualitative Assessment and Risk of Bias

All of the studies evaluated subsamples using cross-sectional methods (13–18). The quality of measurements described in the articles is shown in Table 2. None of the studies evaluated had a high risk of bias. However, some studies reduced the methodological quality due to issues reported in the assessment criteria such as inclusion criteria, subjects of study, exposure measure, outcomes measure, and statistical analysis.

**Table 2:** Quality assessment and risk of bias according to Critical Appraisal Tool (Joanna Briggs Institute, 2017).

	**Rohani et al. (13)**	**Sermet Elbay et al. (14)**	**Arman et al. (15)**	**Nascimento et al. (16)**	**Carvalho et al. (17)**	**Lurie et al. (18)**
Were the criteria for inclusion in the sample clearly defined?	YES	YES	UNCLEAR	YES	YES	YES
Were the study subjects and the setting described in detail?	YES	YES	UNCLEAR	UNCLEAR	UNCLEAR	UNCLEAR
Was the exposure measured in a valid and reliable way?	YES	NO	YES	YES	YES	YES
Were objective, standard criteria used for measurement of the condition?	YES	NO	YES	NO	YES	NO
Were confounding factors identified?	YES	YES	YES	YES	YES	NO
Were strategies to deal with confounding factors stated?	NO	YES	YES	NO	YES	NO
Were the outcomes measured in a valid and reliable way?	YES	NO	YES	YES	YES	YES
Was appropriate statistical analysis used?	YES	YES	YES	UNCLEAR	YES	YES

Two studies included were classified with low risk of bias (15, 17) and the others were classified with a moderate risk of bias (13, 14, 16, 18) (Table 2). In addition, some methodological problems were detected, more specifically: the lack of clarity in inclusion criteria (15); the presence of confounding factors such as smoking habits or other oral dysfunction (13–17); and the lack of clarity regarding stress evaluation (14, 16, 18).

### Meta-Analysis and Certainty of the Evidence

**Figure 2:**
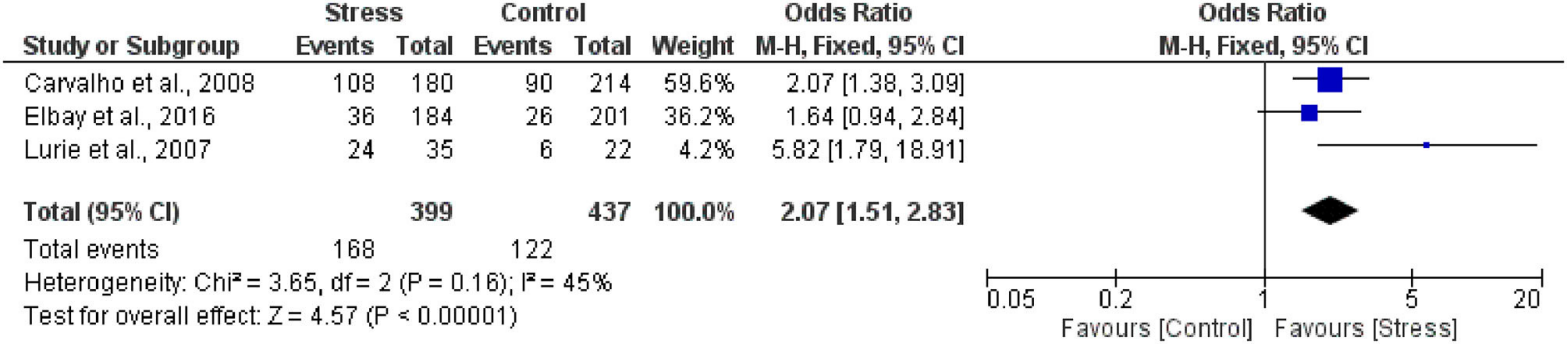
Forest plot of association between stress and bruxism.

Three studies were included in the meta-analysis (14, 15, 17). Three studies were not included in the meta-analysis due to a lack of data in the control group (13, 16, 18) (Figure 2).

The overall heterogeneity was *I*^2^ = 45% (*p* = 0.16) (moderate heterogeneity). The total individuals that in the case group (*n* = 399), 42.1% (*n* = 168), presented bruxism, while 27.9% (*n* = 122) of the total of individuals in the control group (*n* = 437) presented bruxism. Stress and bruxism are positively related once stressed people presented a 97% higher chance to present bruxism (OR 2.07 [1.51, 2.83], *p* < 0.00001), with low certainty of evidence (Table 3).

**Table 3:** The certainty of evidence: association between stress and bruxism.

**Certainty assessment**							**Summary of findings**				
**Participants (studies) Follow up**	**Risk of bias**	**Inconsistency**	**Indirectness**	**Imprecision**	**Other considerations**	**Overall certainty of the evidence**	**Study event rates (%)**		**Relative effect (95% CI)**	**Anticipated absolute effects**	
							**Control**	**With stress**		**Risk control**	**Risk difference with stress**
836 (3 observational studies)	Not serious	Not serious	Not serious	Serious^a^	Strong association	⊕⊕ LOW	122/437 (27.9%)	168/399 (42.1%)	OR 2.07 (1.51–2.83)	279 per 1,000	166 more per 1,000 (from 90 more to 244 more)

*CI, confidence interval; OR, odds ratio*.

a *The upper limit of the confidence interval is >25% of the OR*.

The sensitivity analysis showed that the exclusion of studies with some type of risk of bias did not influenced in overall significance. Besides serious problems in “imprecision” the overall results presented strong association, so, the certainty of evidence was classified as low.

## Discussion

In this systematic review, we gather evidence of the association between stress and bruxism, confirmed in the quantitative analysis. However, this association proved to have a low heterogeneity and a low certainty of evidence due to the elected studies' experimental design.

In this review, six of the chosen studies showed a comparable association of bruxism in patients with stress. The evaluation parameters of stress are related to validated or non-validated questionnaires to determine stress levels. Among these validated questionnaires, the oldest is the Perceived Stress Scale (24), 12-item General Health Questionnaire (25), and the most recent is the Stress Symptoms Inventory (SSI) (26) and non-validated questionnaires about stress events/experience (14, 15, 18). The Perceived Stress Scale (24) consists of one of the most cited instruments in the literature for stress assessment. It is known that stress diagnosis is a subjective criterion correlated to the answer of questionnaires with pre-established scores and scales.

Although the selected studies deal with different types of stress. Two studies assessed emotional stress (16, 17); two studies evaluated psychological stress (13, 18) and two studies evaluated stress in life events or experience (14, 15). Stress is manifested through physiological functions, performance, behavior, and subjective symptoms (27). In these cases, the stress has been associated with several altered cognitive function findings, such as poor processing speed, defective executive functioning, and memory deficits (28). Emotional stress has also been associated with risk factors for cardiovascular dysfunction (3), immune system functions (29), endocrine system (30) and in the stomatognathic system (31).

Another kind of stress evaluated by the included studies was psychosocial stress. This type of stress is induced by situations of social threat, including social evaluation, social exclusion, and achievement situations claiming goal-directed performance (32). Stressful life events could induce a series of psychological changes (27). Psychological and psychosomatic symptoms are related to stress in occupational exposure, anxiety, and depression, financial problems, and periodic headaches, and oral dysfunctions (33).

Bruxism functions as a kind of perpetual motion machine, as intensifying symptoms resulting from an organism's abnormal functioning increase a feeling of being stressed, and in consequence, lead to an increased muscle tone and teeth grinding (34). It is generally accepted that stressful situations and mental diseases conduct to the development of occlusal parafunction and temporomandibular disorders without being the only cause (35). Several studies reported that bruxism, one of the most common parafunctional habits, has psychosocial, emotional, and psychological as a risk factor triggering bruxism.

Bruxism is a repetitive masticatory muscle activity characterized by clenching or grinding of the teeth and/or by bracing or thrusting of the mandible. That is specified as either sleep bruxism or awake bruxism, depending on its circadian phenotype (36). The etiology of bruxism is multifactorial. The anatomy, morphology, and dental occlusion are linked to bruxism (36). Early diagnosis of bruxism is of great importance both for its treatment and for its prevention. The diagnosis must focus on identifying the signs and symptoms reported by the patient or the dentist during a clinical examination (37).

In the present study, the selected articles evaluated bruxism without distinction of types, such as awake or sleep bruxism. This is because the selected articles had their publication year before the new classification on bruxism that advocates this subdivision (5). Therefore, the selected articles encompass a more generalized concept. Three studies evaluated bruxism with clinical examination, teeth clenching and grinding sounds, muscle pain, and tooth wear, the most common evidence of bruxism (16–18). Two studies assessed this condition using clinical examination and validated questionnaires (13, 14). The last one was evaluated with a questionnaire about systemic conditions, including bruxism (15).

The leading cause of bruxism has not been determined but is held to involve multiple factors (38). The risk factors are smoking (39), gastroesophageal reflux disease (40), sleep apnea syndrome (41), genetic and behavior (37), anxiety (42), alcohol excesses (43), depression (44). This systematic review shows that, despite other causal factors, the various types of stress may modulate bruxism.

Considering the problems due to the lack of data in the control group, three studies were excluded from the meta-analysis (13, 16, 17). Furthermore, a meta-analysis was performed. The data of meta-analyses revealed that stressed adults presented 2.07 more chances to present bruxism. Also, a low heterogeneity (*I*^2^ = 45%, *p* = 0.16) was observed in this evidence. In addition to problems with lack of data, failures were detected in the existence of confounding factors resulting from unpaired sample characteristics such as smoking, systemic diseases, types of works.

The level of evidence evaluation through GRADE assessment showed a low level in our analysis. This low certainty of evidence found in this systematic review is related to cross-sectional studies evaluated since the methodologies inconsistencies present discrepancies in sample size, use of non-validated analysis tools, and the available data.

As the strengths of this review, we pointed out the inclusion of observational studies regardless of the age or stress evaluation methods adopted by the studies. This review describes the results and the quality of the current evidence on the topic, suggesting an association between bruxism and stress and highlighting the future direction for future research. Despite the low level of certainty observed in GRADE, it is essential to note that, in the quantitative data, the selected studies suggest that patients with stress are very likely to present bruxism. These results must be questionable due to the substantial heterogeneity detected and the inconsistency of the evidence. Therefore, the results suggest that more studies are needed to establish a high certainty of evidence related to stress and bruxism.

## Conclusion

This systematic review suggests a significant association between bruxism in patients stressed, especially in emotional disorders and occupational exposures. Our meta-analysis shows low heterogeneity between the studies due to a low level of evidence, which resulted in limitations of the bruxism evaluation parameters and lack of methodological criteria. Therefore, more studies with representative samples and other clinical assessments on stress and bruxism are necessary to establish this possible association.

## Data Availability Statement

The original contributions presented in the study are included in the article/Supplementary Material, further inquiries can be directed to the corresponding author.

## Author Contributions

VC, CS, LM, and RL: study concept and design. VC, YN, DF, and MM: analysis and interpretation of data. VC, RS-R, and NF: preparation of the manuscript. LM and RL: critical revision of the manuscript. All authors contributed to the article and approved the submitted version.

## Conflict of Interest

The authors declare that the research was conducted in the absence of any commercial or financial relationships that could be construed as a potential conflict of interest.
