# Supplementary material for: Is There Association Between Stress and Bruxism? A Systematic Review and Meta-Analysis
Source: Front Neurol. 2020 Dec 7;11:590779. doi: 10.3389/fneur.2020.590779 (PMC7793806; doi:10.3389/fneur.2020.590779)
Supplement: Supplementary file 1 [file Data_Sheet_1.docx]

Supplementary Material

**Supplementary Table 1.** PRISMA-P (Preferred Reporting Items for Systematic review and Meta-Analysis Protocols) 2015 checklist (Moher et al., 2015).

| **Section and topic** | **Item No** | **Checklist item** |
| --- | --- | --- |
| **ADMINISTRATIVE INFORMATION** | | |
| Title: |  |  |
| Identification | 1a | Identify the report as a protocol of a systematic review |
| Update | 1b | If the protocol is for an update of a previous systematic review, identify as such |
| Registration | 2 | If registered, provide the name of the registry (such as PROSPERO) and registration number |
| Authors: |  |  |
| Contact | 3a | Provide name, institutional affiliation, e-mail address of all protocol authors; provide physical mailing address of corresponding author |
| Contributions | 3b | Describe contributions of protocol authors and identify the guarantor of the review |
| Amendments | 4 | If the protocol represents an amendment of a previously completed or published protocol, identify as such and list changes; otherwise, state plan for documenting important protocol amendments |
| Support: |  |  |
| Sources | 5a | Indicate sources of financial or other support for the review |
| Sponsor | 5b | Provide name for the review funder and/or sponsor |
| Role of sponsor or funder | 5c | Describe roles of funder(s), sponsor(s), and/or institution(s), if any, in developing the protocol |
| **INTRODUCTION** | | |
| Rationale | 6 | Describe the rationale for the review in the context of what is already known |
| Objectives | 7 | Provide an explicit statement of the question(s) the review will address with reference to participants, interventions, comparators, and outcomes (PICO) |
| **METHODS** | | |
| Eligibility criteria | 8 | Specify the study characteristics (such as PICO, study design, setting, time frame) and report characteristics (such as years considered, language, publication status) to be used as criteria for eligibility for the review |
| Information sources | 9 | Describe all intended information sources (such as electronic databases, contact with study authors, trial registers or other grey literature sources) with planned dates of coverage |
| Search strategy | 10 | Present draft of search strategy to be used for at least one electronic database, including planned limits, such that it could be repeated |
| Study records: |  |  |
| Data management | 11a | Describe the mechanism(s) that will be used to manage records and data throughout the review |
| Selection process | 11b | State the process that will be used for selecting studies (such as two independent reviewers) through each phase of the review (that is, screening, eligibility and inclusion in meta-analysis) |
| Data collection process | 11c | Describe planned method of extracting data from reports (such as piloting forms, done independently, in duplicate), any processes for obtaining and confirming data from investigators |
| Data items | 12 | List and define all variables for which data will be sought (such as PICO items, funding sources), any pre-planned data assumptions and simplifications |
| Outcomes and prioritization | 13 | List and define all outcomes for which data will be sought, including prioritization of main and additional outcomes, with rationale |
| Risk of bias in individual studies | 14 | Describe anticipated methods for assessing risk of bias of individual studies, including whether this will be done at the outcome or study level, or both; state how this information will be used in data synthesis |
| Data synthesis | 15a | Describe criteria under which study data will be quantitatively synthesised |
|  | 15b | If data are appropriate for quantitative synthesis, describe planned summary measures, methods of handling data and methods of combining data from studies, including any planned exploration of consistency (such as I^2^, Kendall’s τ) |
|  | 15c | Describe any proposed additional analyses (such as sensitivity or subgroup analyses, meta-regression) |
|  | 15d | If quantitative synthesis is not appropriate, describe the type of summary planned |
| Meta-bias(es) | 16 | Specify any planned assessment of meta-bias(es) (such as publication bias across studies, selective reporting within studies) |
| Confidence in cumulative evidence | 17 | Describe how the strength of the body of evidence will be assessed (such as GRADE) |

**Supplementary Table 2.** Terms used on databases searches.

| Database | Search format |
| --- | --- |
| PUBMED  N=313 | (((((((((((((((life stress[Title/Abstract]) OR suffering[Title/Abstract]) OR psychological stress[Title/Abstract]) OR emotional stress[Title/Abstract]) OR physiological stress[Title/Abstract]) OR metabolic stress[Title/Abstract]) OR biological stress[Title/Abstract]) OR cortisol[Title/Abstract]) OR hydrocortisone[MeSH Terms]) OR hydrocortisone[Title/Abstract]) OR chronic* stress[Title/Abstract]) OR stress*[Title/Abstract]) OR stress psychological[MeSH Terms]) OR stress psychological[Title/Abstract]) OR stress physiological[MeSH Terms]) OR stress physiological[Title/Abstract] AND (((((bruxism[MeSH Terms]) OR bruxism[Title/Abstract]) OR sleep bruxism[MeSH Terms]) OR sleep bruxism[Title/Abstract]) OR nocturnal bruxism[Title/Abstract]) OR sleep related bruxism[Title/Abstract] |
| SCOPUS  N=705 | ( ( TITLE-ABS-KEY ( hydrocortisone ) OR TITLE-ABS-KEY ( cortisol ) OR TITLE-ABS-KEY ( "Stress, Psychological" ) OR TITLE-ABS-KEY ( "Life Stress" ) OR TITLE-ABS-KEY ( "Psychological Stress" ) OR TITLE-ABS-KEY ( "Suffering" ) OR TITLE-ABS-KEY ( "Emotional Stress" ) OR TITLE-ABS-KEY ( "Stress, Physiological" ) OR TITLE-ABS-KEY ( "Physiological Stress" ) OR TITLE-ABS-KEY ( "Metabolic Stress" ) OR TITLE-ABS-KEY ( "Biological Stress" ) OR TITLE-ABS-KEY ( "Chronic* stress" ) OR TITLE-ABS-KEY ( stress* ) ) ) AND ( TITLE-ABS-KEY ( bruxism ) OR TITLE-ABS-KEY ( "sleep bruxism" ) OR TITLE-ABS-KEY ( "nocturnal bruxism" ) OR TITLE-ABS-KEY ( "sleep related bruxism" ) ) ) |
| COCHRANE  N=30 | (Hydrocortisone):ti,ab,kw OR (Cortisol):ti,ab,kw OR ("Stress, Psychological"):ti,ab,kw OR ("Life Stress"):ti,ab,kw OR ("Psychological Stress"):ti,ab,kw OR (Suffering):ti,ab,kw OR ("Emotional Stress"):ti,ab,kw OR ("Stress, Physiological"):ti,ab,kw OR ("Physiological Stress"):ti,ab,kw OR ("Metabolic Stress"):ti,ab,kw OR ("Biological Stress"):ti,ab,kw OR (Chronic$ stress):ti,ab,kw OR (Stress$):ti,ab,kw AND (bruxism):ti,ab,kw OR ("sleep related bruxism"):ti,ab,kw OR ("sleep bruxism"):ti,ab,kw OR ("nocturnal bruxism"):ti,ab,kw |
| WEB OF SCIENCE  N=327 | TS=(Hydrocortisone OR Cortisol) OR “Stress, Psychological" OR "Life Stress" OR "Psychologic Stress" OR "Psychological Stress" OR Suffering OR "Emotional Stress" OR "Stress, Physiological" OR "Physiological Stress" OR "Metabolic Stress" OR "Biological Stress" OR Chronic* stress OR stress*) AND TS= (bruxism OR "sleep bruxism" OR "nocturnal bruxism" OR "sleep related bruxism") |
| LILACS  N=69 | Hydrocortisone OR Cortisol OR "Stress, Psychological" OR "Life stress" OR "Psychological Stress" OR Suffering OR "Emotional Stress" OR "Stress, Physiological" OR "Physiological Stress" OR "Metabolic Stress" OR "Biological Stress" OR Chronic* stress OR stress* AND bruxism OR “sleep bruxism” OR “nocturnal bruxism” OR “sleep related bruxism” |
| GOOGLE SCHOLAR  N=14 | Bruxism AND Hydrocortisone AND Stress, Psychological AND Stress, Physiological AND NOT books AND NOT systematic review AND NOT reviews |
| OPEN GREY  N=0 | Bruxism AND Hydrocortisone AND “Stress, Psychological” AND “Stress, Physiological” |

**Supplementary Table 3.** Analytical cross-sectional studies Critical Appraisal Tool

| **Guideline** | **Description** |
| --- | --- |
| Were the criteria for inclusion in the sample clearly defined? | The authors should provide clear inclusion and exclusion criteria that they developed prior to recruitment of the study participants. The inclusion/exclusion criteria should be specified (e.g., risk, stage of disease progression) with sufficient detail and all the necessary information critical to the study. |
|  |  |
|  |  |
|  |  |
|  |  |
| Were the study subjects and the setting described in detail? |  |
|  |  |
|  | The study sample should be described in sufficient detail so that other researchers can determine if it is comparable to the population of interest to them. The authors should provide a clear description of the population from which the study participants were selected or recruited, including demographics, location, and time period. |
|  |  |
|  |  |
| Was the exposure measured in a valid and reliable way? | The study should clearly describe the method of measurement of exposure. Assessing validity requires that a 'gold standard' is available to which the measure can be compared. The validity of exposure measurement usually relates to whether a current measure is appropriate or whether a measure of past exposure is needed. Reliability refers to the processes included in an epidemiological study to check repeatability of measurements of the exposures. These usually include intra-observer reliability and interobserver reliability. |
|  |  |
|  |  |
|  |  |
| Were objective, standard criteria used for measurement of the condition? |  |
|  | It is useful to determine if patients were included in the study based on either a specified diagnosis or definition. This is more likely to decrease the risk of bias. Characteristics are another useful approach to matching groups, and studies that did not use specified diagnostic methods or definitions should provide evidence on matching by key characteristics. |
|  |  |
|  |  |
| Were confounding factors identified? |  |
|  | Confounding has occurred where the estimated intervention exposure effect is biased by the presence of some difference between the comparison groups (apart from the exposure investigated/of interest). Typical confounders include baseline characteristics, prognostic factors, or concomitant exposures (e.g. smoking). A confounder is a difference between the comparison groups and it influences the direction of the study results. A high quality study at the level of cohort design will identify the potential confounders and measure them (where possible). This is difficult for studies where behavioral, attitudinal or lifestyle factors may impact on the results. |
|  |  |
|  |  |
| Were strategies to deal with confounding factors stated? |  |
|  | Strategies to deal with effects of confounding factors may be dealt within the study design or in data analysis. By matching or stratifying sampling of participants, effects of confounding factors can be adjusted for. When dealing with adjustment in data analysis, assess the statistics used in the study. Most will be some form of multivariate regression analysis to account for the confounding factors measured |
|  |  |
|  |  |
|  |  |
| Were the outcomes measured in a valid and reliable way? | Read the methods section of the paper. If for e.g. lung cancer is assessed based on existing definitions or diagnostic criteria, then the answer to this question is likely to be yes. If lung cancer is assessed using observer reported, or self-reported scales, the risk of over- or underreporting is increased, and objectivity is compromised. Importantly, determine if the measurement tools used were validated instruments as this has a significant impact on outcome assessment validity. Having established the objectivity of the outcome measurement (e.g. lung cancer) instrument, it’s important to establish how the measurement was conducted. Were those involved in collecting data trained or educated in the use of the instrument/s? (e.g. radiographers). If there was more than one data collector, were they similar in terms of level of education, clinical or research experience, or level of responsibility in the piece of research being appraised? |
| Was appropriate statistical analysis used? | As with any consideration of statistical analysis, consideration should be given to whether there was a more appropriate alternate statistical method that could have been used. The methods section should be detailed enough for reviewers to identify which analytical techniques were used (in particular, regression or stratification) and how specific confounders were measured. For studies utilizing regression analysis, it is useful to identify if the study identified which variables were included and how they related to the outcome. If stratification was the analytical approach used, were the strata of analysis defined by the specified variables? Additionally, it is also important to assess the appropriateness of the analytical strategy in terms of the assumptions associated with the approach as differing methods of analysis are based on differing assumptions about the data and how it will respond. |

**Supplementary Table 4.** Articles excluded after reading in full and reasons for exclusions.

| Reference | Reason for exclusion |
| --- | --- |
| Pawlak, Ł., Suliborski, B., Sajewicz-Rosiak, M., Romańczuk, K., & Split, W. (2008). Psychosocial stress influence on parafunction and oromandibular dysfunction (OMD) prevalence among adolescents. Clin Exp Med Lett, 49(3), 179-83. | Absence of bruxism evaluation |
| Anna, S., Joanna, K., Teresa, S., Maria, G., & Aneta, W. (2015). The influence of emotional state on the masticatory muscles function in the group of young healthy adults. *BioMed research international*, *2015*. | Absence of bruxism evaluation |
| Vanderas, A. P. (1996). Synergistic effect of malocclusion and oral parafunctions on craniomandibular dysfunction in children with and without unpleasant life events. Journal of oral rehabilitation, 23(1), 61-65. | Absence of bruxism evaluation |
| Vanderas, A. P., Menenakou, M., Kouimtzis, T. H., & Papagiannoulis, L. (1999). Urinary catecholamine levels and bruxism in children. Journal of oral rehabilitation, 26(2), 103-110. | Independent evaluation of bruxism and stress with no correlation among them |
| Ahlberg, K., Ahlberg, J., Könönen, M., Partinen, M., Hublin, C., & Savolainen, A. (2005). Reported bruxism and restless legs syndrome in media personnel with or without irregular shift work. Acta Odontologica Scandinavica, 63(2), 94-98. | Absence of stress evaluation |
| Pingitore, G., Chrobak, V., & Petrie, J. (1991). The social and psychologic factors of bruxism. The journal of prosthetic dentistry, 65(3), 443-446. | Absence of control group |
| Rao, S. K., Bhat, M., & David, J. (2011). Work, stress, and diurnal bruxism: a pilot study among information technology professionals in Bangalore City, India. International journal of dentistry, 2011. | Absence of control group |
| Vidaković, B., Grgurević, J., & Sonicki, Z. (2014). Myofascial pain in war veterans with post-traumatic stress disorder. Journal of Musculoskeletal Pain, 22(2), 182-186. | Presence of anxiety related disorder |
| Kamiński, B., Pawlak, Ł., & Split, W. The effect of compulsory military service on the prevalence of masticatory parafunctions in the study subjects. Clin Exp Med Lett 2009; 50(1)55-58. | Control group is the same as the exposed group, in different analysis times |
